# Supplementary figures and images for: mTOR-dependent translation drives tumor infiltrating CD8+ effector and CD4+ Treg cells expansion
Source: eLife. 2021 Nov 17;10:e69015. doi: 10.7554/eLife.69015 (PMC8598161; doi:10.7554/eLife.69015)

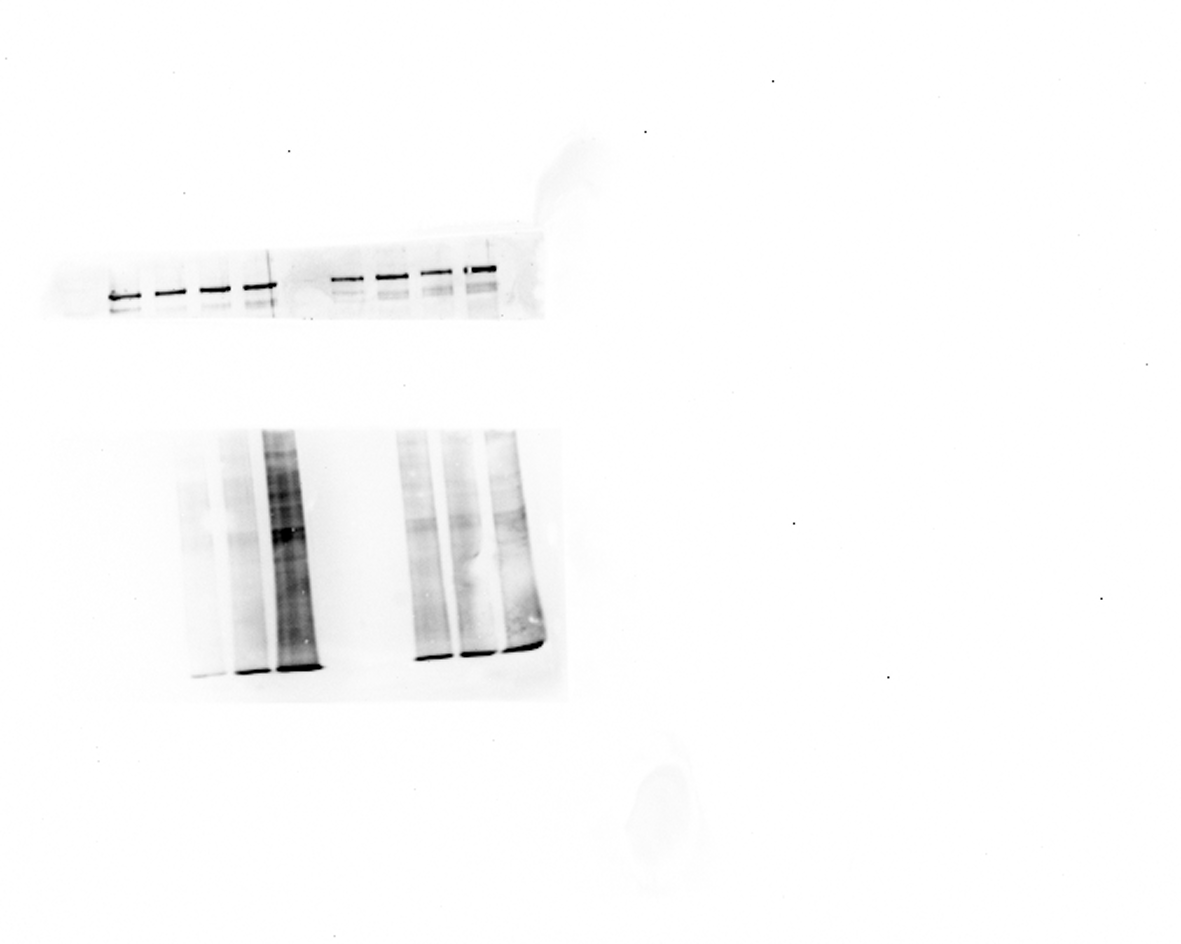

Supplement: Figure 1—source data 1. [file elife-69015-fig1-data1.zip › Source data 1 /Figure 1_source data 1.tiff]

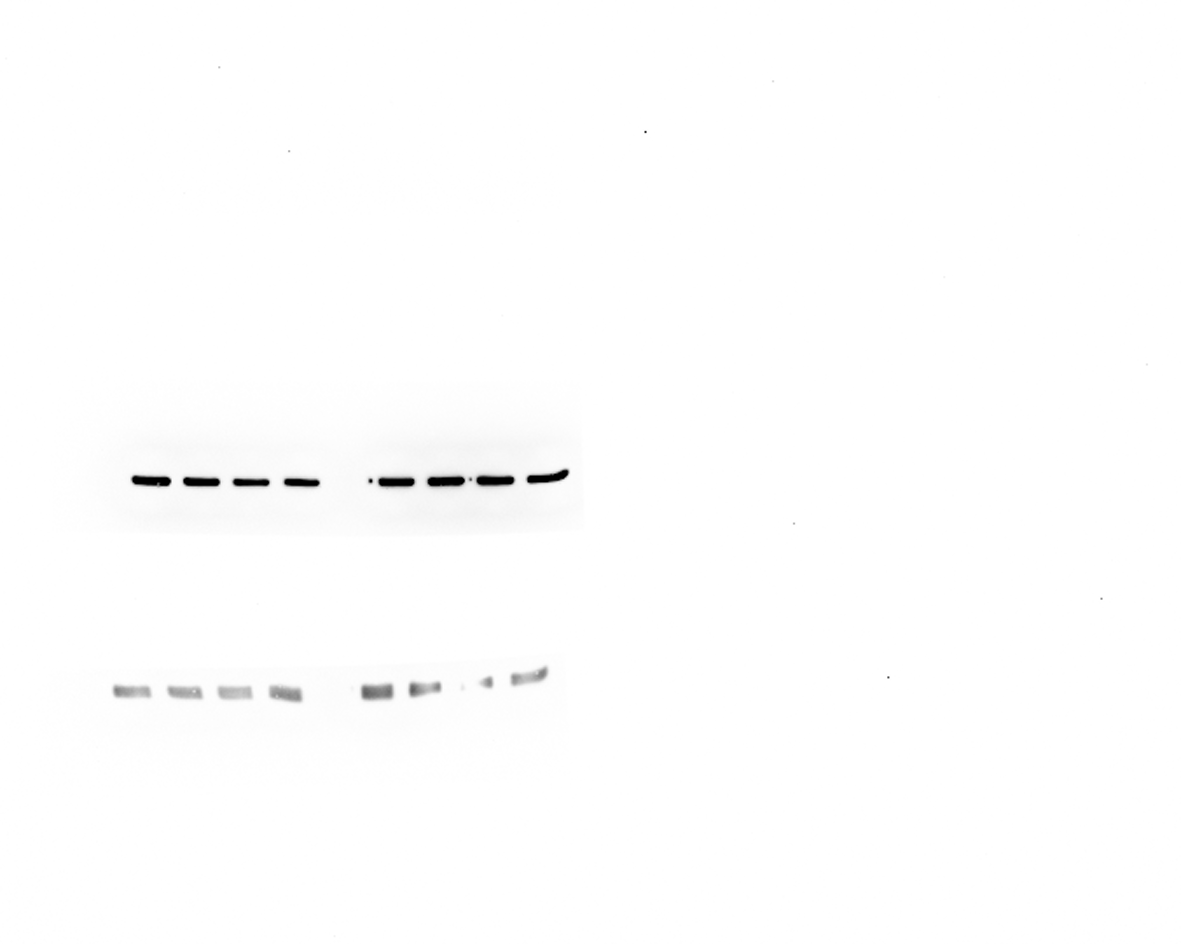

Supplement: Figure 1—source data 1. [file elife-69015-fig1-data1.zip › Source data 1 /Figure 1_source data 8.tif]

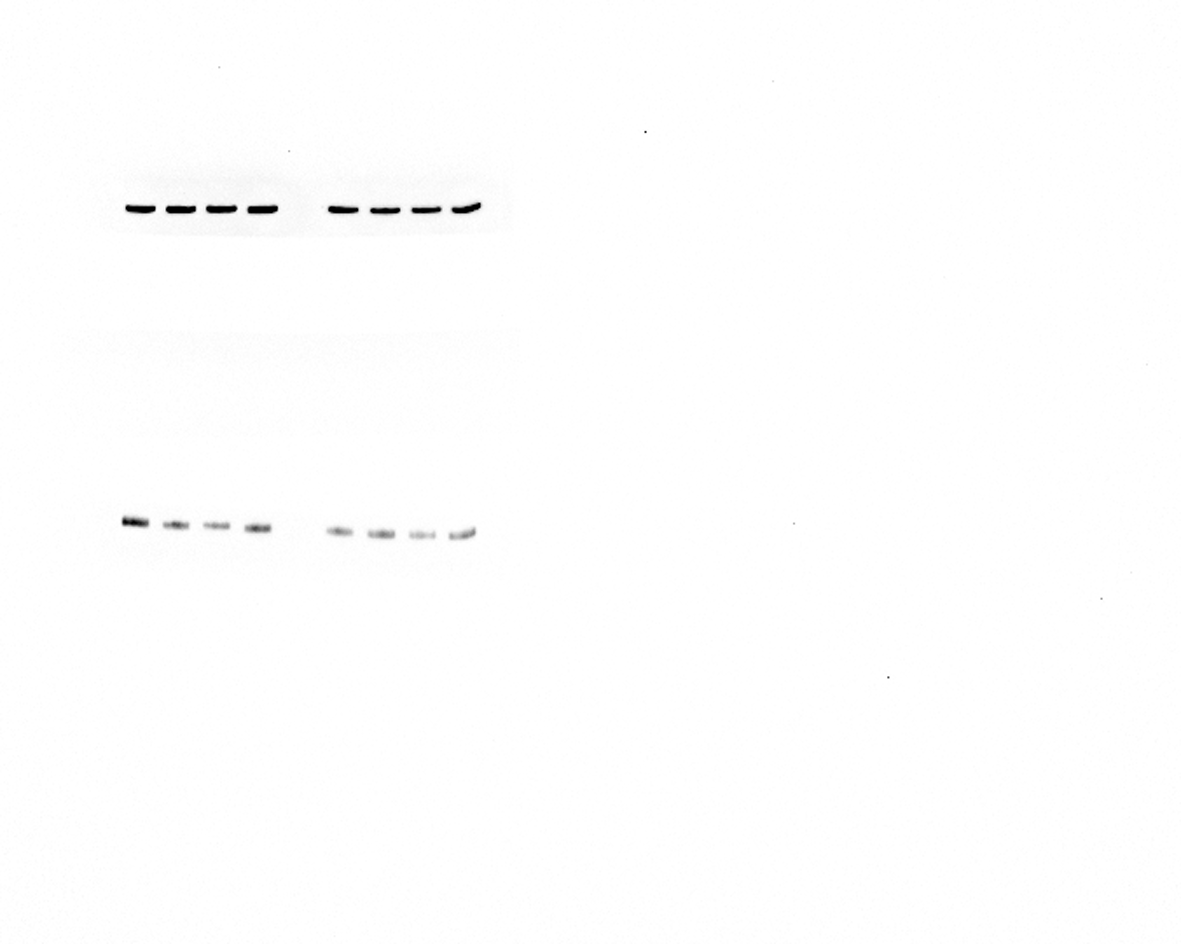

Supplement: Figure 1—source data 1. [file elife-69015-fig1-data1.zip › Source data 1 /Figure 1_source data 7.tiff]

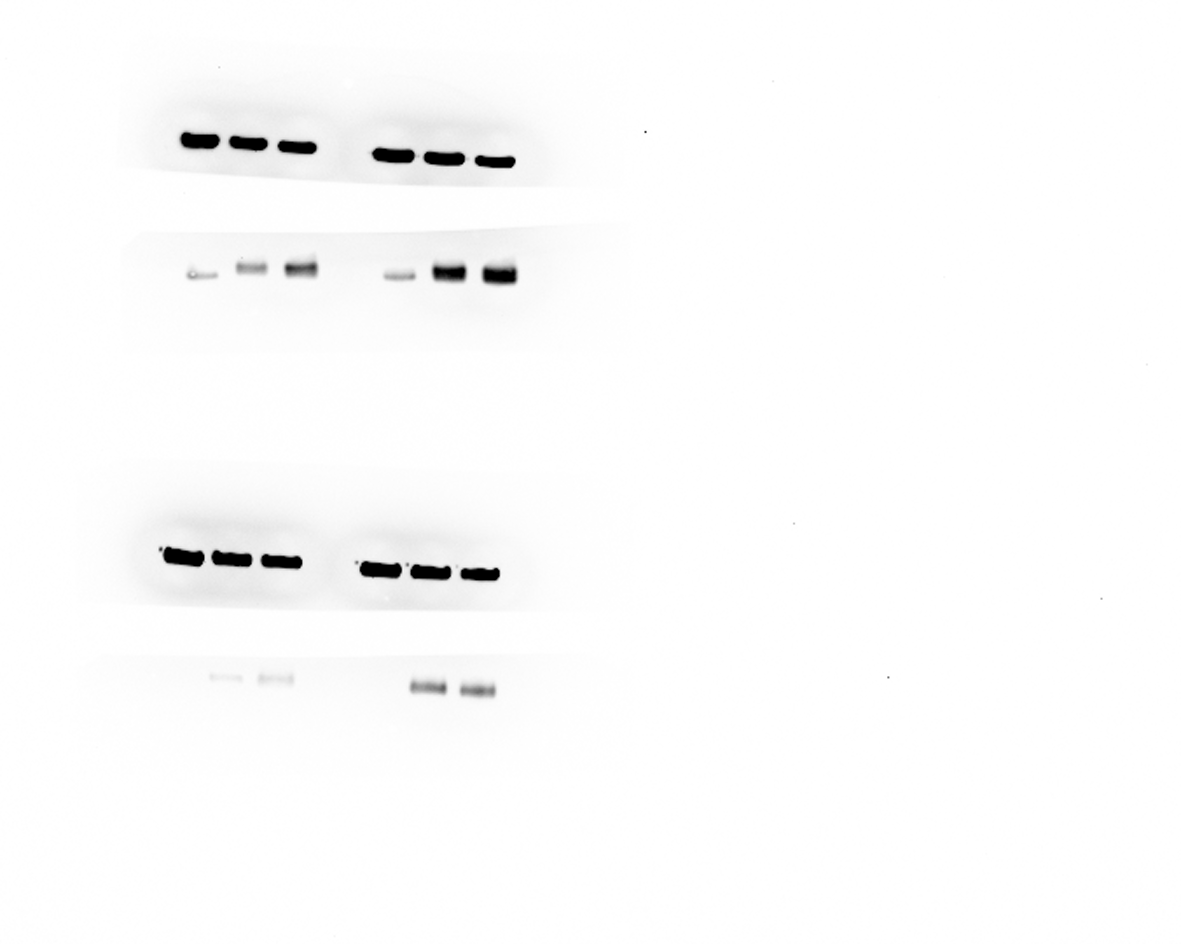

Supplement: Figure 1—source data 1. [file elife-69015-fig1-data1.zip › Source data 1 /Figure 1_source data 4.tiff]

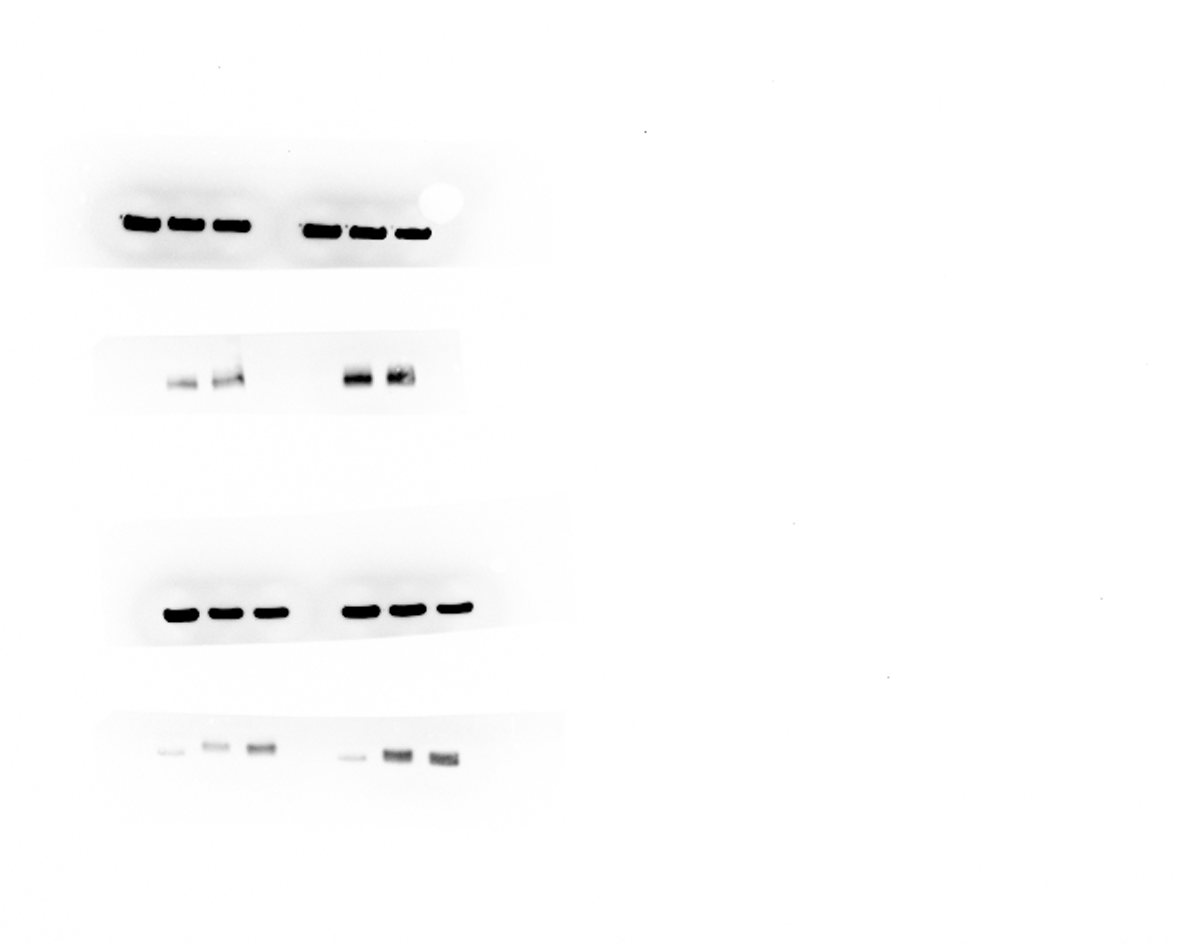

Supplement: Figure 1—source data 1. [file elife-69015-fig1-data1.zip › Source data 1 /Figure 1_source data 5.tiff]

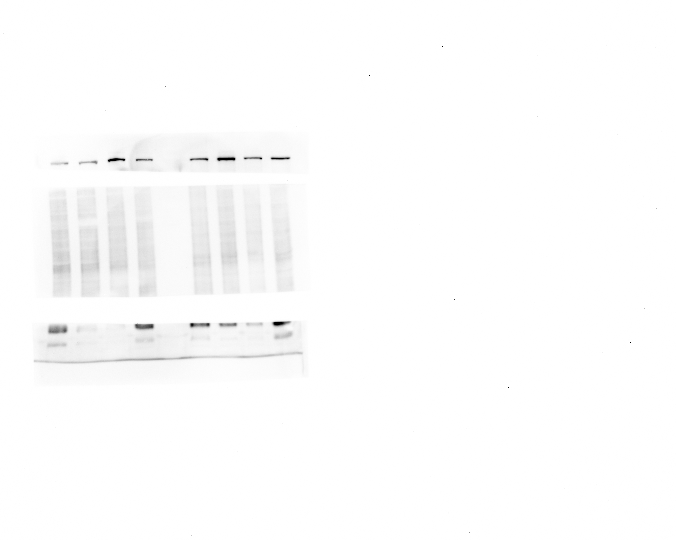

Supplement: Figure 1—source data 1. [file elife-69015-fig1-data1.zip › Source data 1 /Figure 1_source data 6.tif]

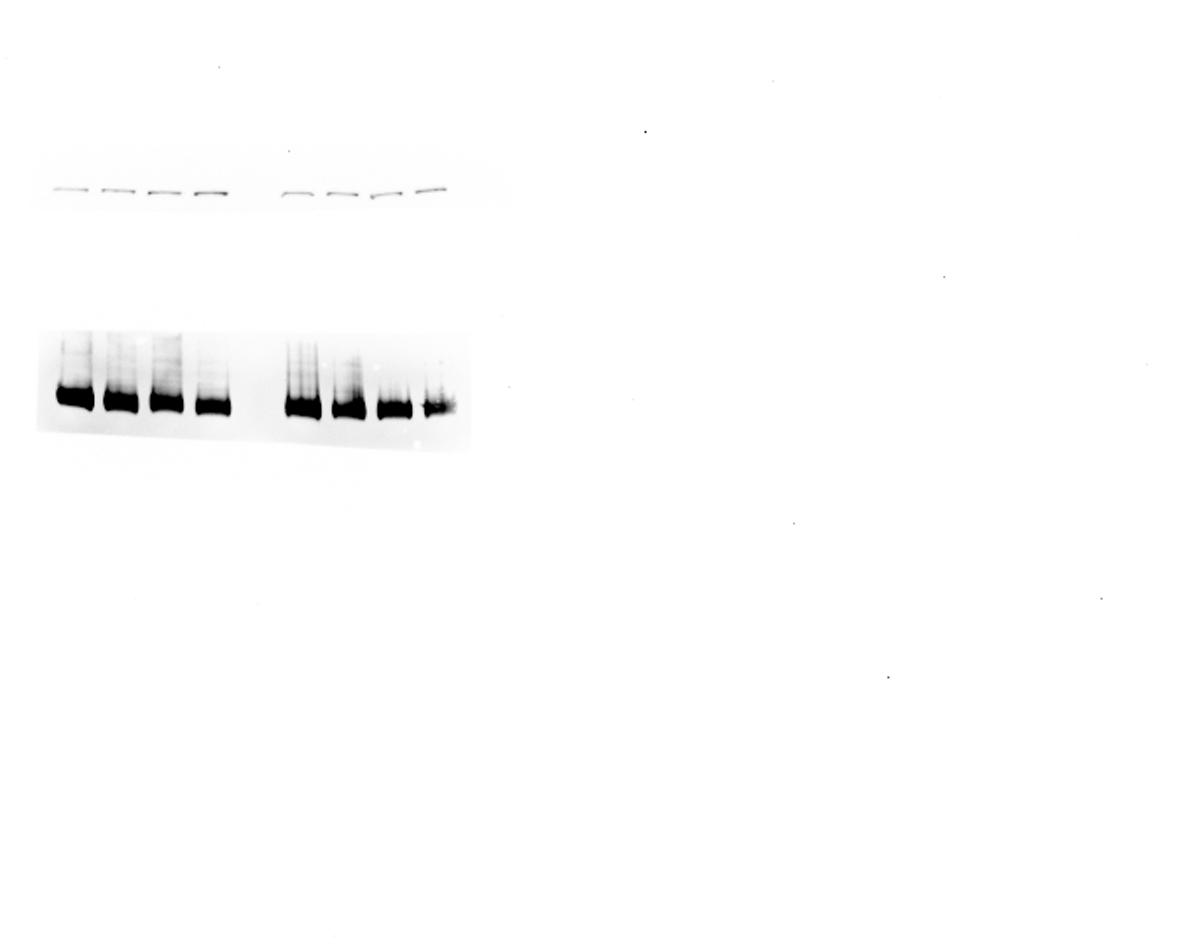

Supplement: Figure 1—source data 1. [file elife-69015-fig1-data1.zip › Source data 1 /Figure 1_source data 9.tiff]

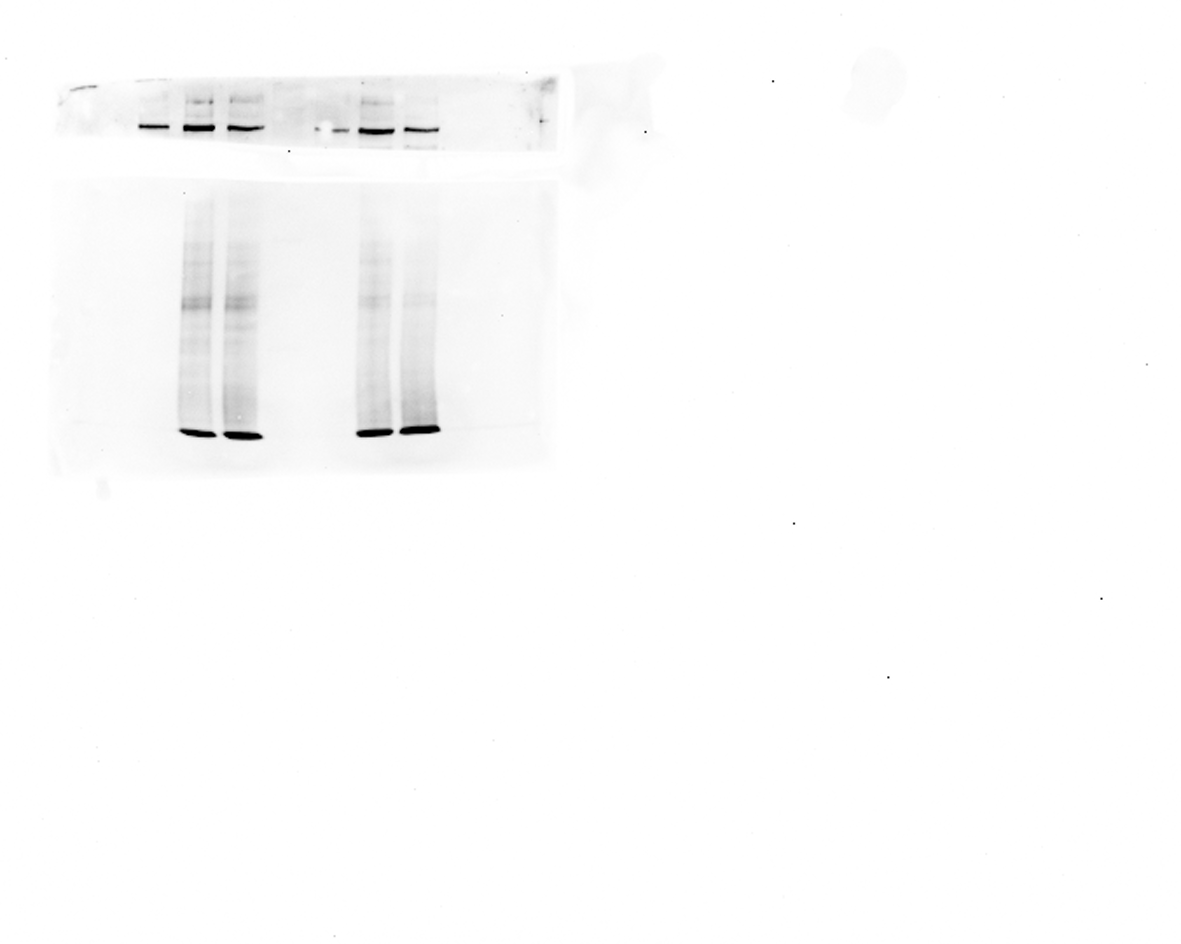

Supplement: Figure 1—source data 1. [file elife-69015-fig1-data1.zip › Source data 1 /Figure 1_source data 2.tiff]

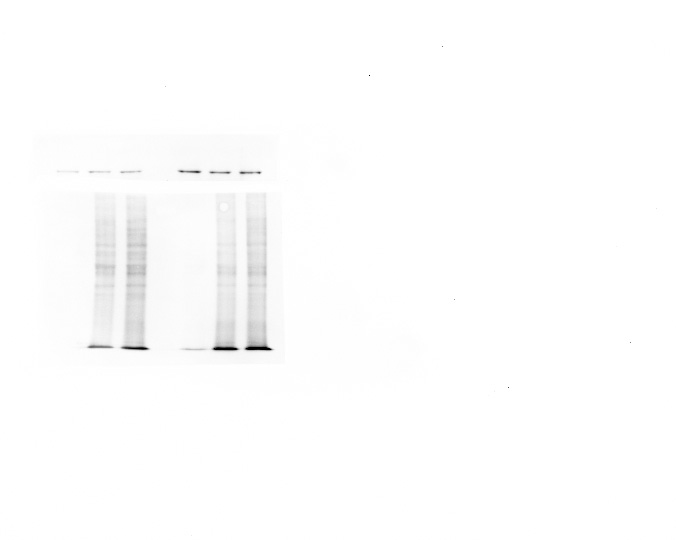

Supplement: Figure 1—source data 1. [file elife-69015-fig1-data1.zip › Source data 1 /Figure 1_source data 3.tiff]

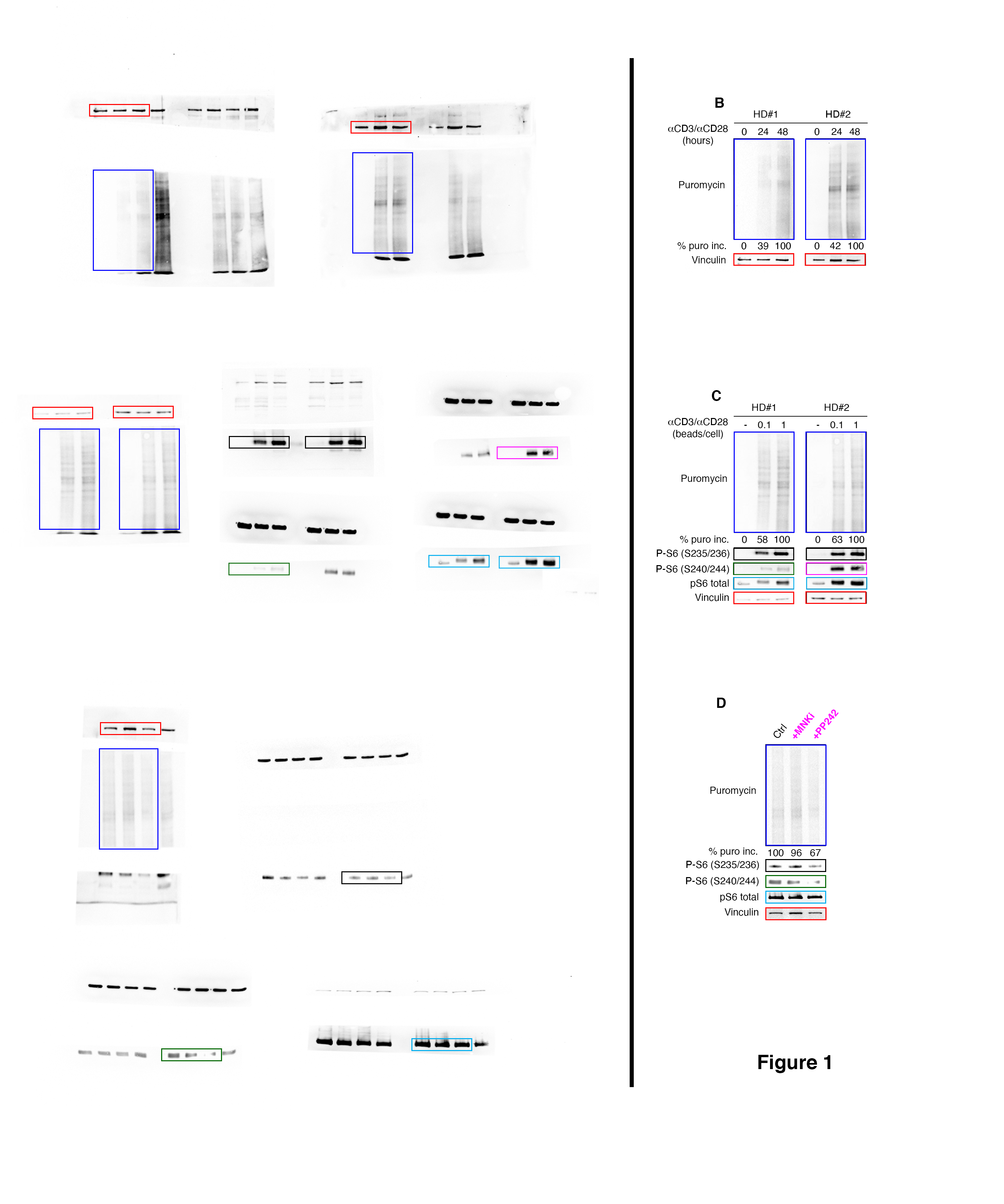

Supplement: Figure 1—source data 2. [file elife-69015-fig1-data2.zip › Source data 2/Figure 1_source data 1.tif]
